# Evaluation of magnetic resonance imaging artifacts caused by fixed orthodontic CAD/CAM retainers—an in vitro study

**DOI:** 10.1007/s00784-020-03450-x

**Published:** 2020-08-12

**Authors:** Christoph Roser, Tim Hilgenfeld, Sinan Sen, Tobias Badrow, Sebastian Zingler, Sabine Heiland, Martin Bendszus, Christopher J. Lux, Alexander Juerchott

**Affiliations:** 1 Department of Orthodontics and Dentofacial Orthopedics, Heidelberg University Hospital, Im Neuenheimer Feld 400, 69120 Heidelberg, Germany; 2 Department of Neuroradiology, Heidelberg University Hospital, Im Neuenheimer Feld 400, 69120 Heidelberg, Germany

**Keywords:** Orthodontics, Retainer, CAD/CAM, MRI, Artifacts

## Abstract

**Objectives:**

Magnetic resonance imaging (MRI) image quality can be severely impaired by artifacts caused by fixed orthodontic retainers. In clinical practice, there is a trend towards using computer-aided design/computer-aided manufacturing (CAD/CAM) retainers. This study aimed to quantify MRI artifacts produced by these novel CAD/CAM retainers.

**Material and methods:**

Three CAD/CAM retainers and a stainless-steel retainer (“Twistflex”; clinical reference standard) were scanned in vitro at 3-T MRI using a high-resolution 3D sequence. The artifact diameters and three-dimensional artifact volumes (AV) were determined for all mandibular (AV_mand_) and maxillary (AV_max_) retainers. Moreover, the corresponding ratio of artifact volume to retainer volume (AV/RV_mand_, AV/RV_max_) was calculated.

**Results:**

Twistflex caused large artifact volumes (AV_mand_: 13530 mm^3^; AV_max_: 15642 mm^3^; AV/RV_mand_: 2602; AV/RV_max_: 2235). By contrast, artifact volumes for CAD/CAM retainers were substantially smaller: whereas artifact volumes for cobalt–chromium retainers were moderate (381 mm^3^; 394 mm^3^; 39; 31), grade-5 titanium (110 mm^3^; 126 mm^3^; 12; 12) and nickel–titanium (54 mm^3^; 78 mm^3^; 12; 14) both produced very small artifact volumes.

**Conclusion:**

All CAD/CAM retainers caused substantially smaller volumes of MRI artifacts compared to Twistflex. Grade-5 titanium and nickel–titanium CAD/CAM retainers showed the smallest artifact volumes.

**Clinical relevance:**

CAD/CAM retainers made from titanium or nickel–titanium may not relevantly impair image quality in head/neck and dental MRI. Artifacts caused by cobalt–chromium CAD/CAM retainers may mask nearby dental/periodontal structures. In contrast, the large artifacts caused by Twistflex are likely to severely impair diagnosis of oral and adjacent pathologies.

## Introduction

Magnetic resonance imaging (MRI) is an essential, non-ionizing imaging technique that is increasingly being used to diagnose disorders of the head and neck region [1]. Moreover, due to recent technical milestones, use of MRI in dental imaging is also increasing [2–6]. This trend is reflected in promising results from in vitro and in vivo dental MRI studies in the fields of periodontology [7–9], endodontics [10, 11], cariology [12], and implantology [13–15]. Furthermore, MRI could play a key role in orthodontics in the future; recent pilot studies have proven the feasibility of MRI-based cephalometric analysis [16–18]. In particular, the diagnosis of disorders of the oral cavity and maxillofacial area in general can be severely impaired by metal-induced susceptibility artifacts, because the region of interest is near the orthodontic appliance [19–21]. As one of the most common and most severe causes of susceptibility artifacts in this area, orthodontic appliances are particularly problematic [22]. Fixed orthodontic retainers are especially relevant in this regard because they are in situ for much longer than other orthodontic appliances [23]. Therefore, substantial clinical benefit would be gained from gathering precise information on artifacts caused by different types of retainer.

In the course of the digitalization of orthodontics, new retainer designs, materials, and manufacturing procedures have been introduced. Unlike the traditional manufacturing process of conventional retainers, more and more manufacturers are offering computer-aided design/computer-aided manufacturing (CAD/CAM) techniques based on intraoral scans or digitized plaster models. These CAD/CAM retainers differ with regard to the materials (mostly cobalt–chromium, titanium, and nickel–titanium) and production techniques used. For example, it is possible to manufacture retainers using the “laser cutting process,” in which retainers are cut out of a blank by a laser. Furthermore, an additive process can be used for manufacturing. CAM processes, such as milling retainers from a titanium block using a five-axis milling system, are also used. Because lingual CAD/CAM retainers have only been available for a few years, no long-term studies of this type of retainer have yet been performed. However, clinical studies of NiTi CAD/CAM retainers demonstrated that they are superior to conventional “Twistflex” retainers in terms of their positioning accuracy, especially in complex occlusal situations [24, 25]. An in vitro study revealed that CAD/CAM NiTi retainers have better biomechanical properties than conventional Twistflex retainers [26]. Because they offer the practitioner further advantages in addition to those already mentioned, it can therefore be assumed that CAD/CAM retainers will become increasingly popular. For example, conventional impressions do not have to be taken and plaster models do not have to be produced, which reduces material expenses and laboratory work for the dentist. Several studies have examined MRI artifacts caused by orthodontic appliances. Most of these studies have focused on temporary orthodontic appliances (brackets, arches, anchoring appliances) [22, 27–29]. Fixed retainers, however, are in most cases worn intraorally by the patient for their entire lifetime [23]. Accordingly, retainer-associated MRI artifacts are relevant for both younger and older patients and will therefore become an increasingly important consideration for MR imaging in the future. However, removing retainers before an MRI scan can result in unnecessary detrimental outcomes for the patient, such as enamel damage, expense, or an orthodontic relapse [30, 31]. Thus, the material of the retainer and the area of interest should be considered when deciding whether retainers should be removed.

Only a few studies have investigated MRI artifacts caused by fixed retainers, and all of these were based on conventional retainers [32–34]. Moreover, no study has investigated the effect of novel CAD/CAM retainers on MRI artifacts. Therefore, we selected three digitally designed and manufactured retainers made from cobalt–chromium, grade-5 titanium, and nickel–titanium. All retainers were embedded in agar, whose use as a substance for in vitro analysis of artifact volumes is well established [35]. The embedded retainers were scanned using a clinically established 3D sequence to achieve the best possible approximation to clinical reality. The aim was to quantify the volume of MRI artifacts caused by these three CAD/CAM retainers by means of direct comparison with the widely used, stainless-steel, five-stranded Twistflex retainer (clinical reference standard).

## Materials and methods

### Selection and production of retainers

**Fig. 1:**
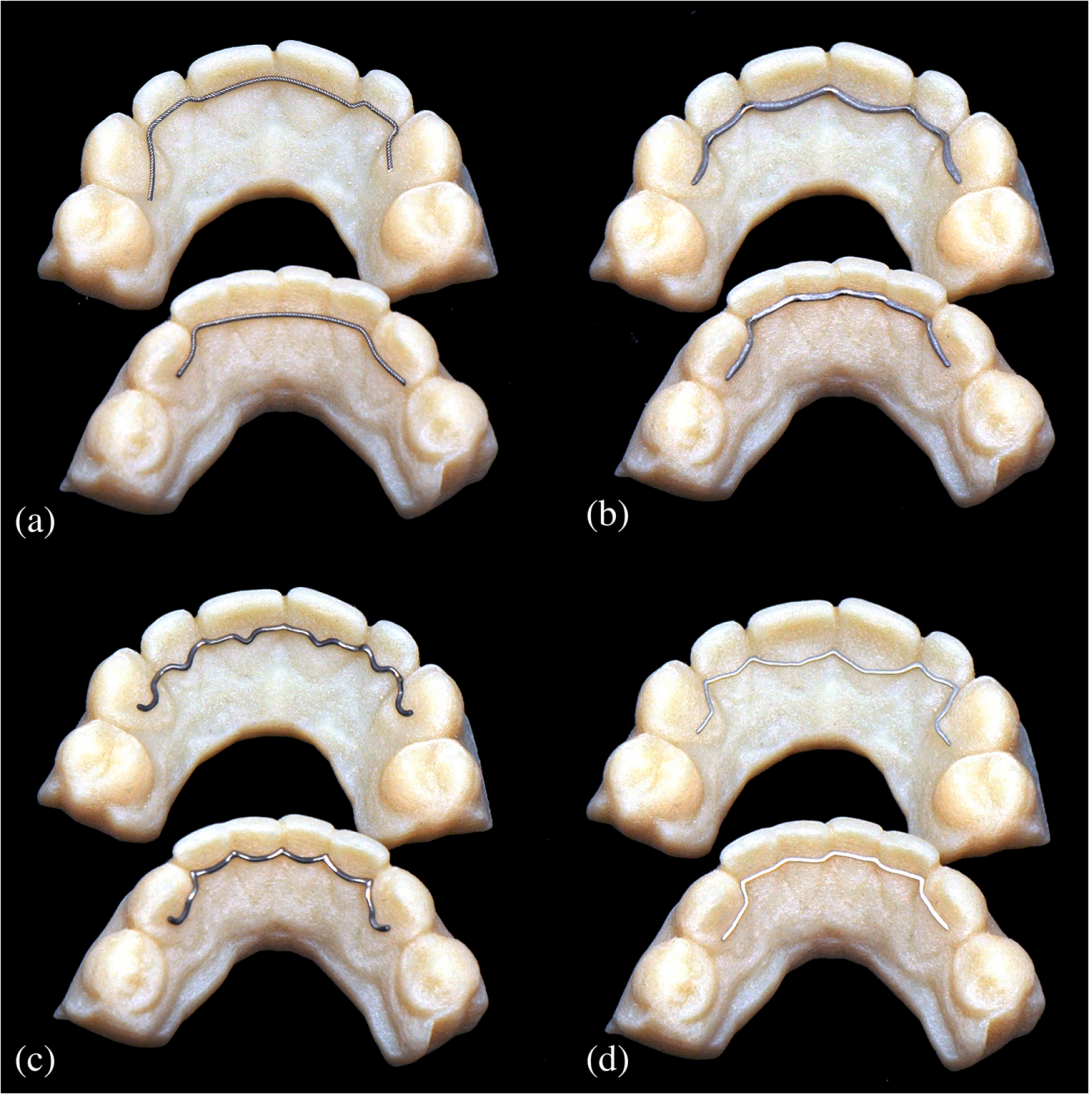
**a–d** Photographs of the retainers used in the study. **a** Twistflex (bent). **b** Cobalt–chromium (CAD/CAM). **c** Grade-5 titanium (CAD/CAM). **d** Nickel–titanium (CAD/CAM). CAD/CAM, computer-aided design/computer-aided manufacturing

Relevant information, such as alloy components of the retainer materials and production methods, were obtained prior to the study from the respective manufacturer (Table 1). For the design of the CAD/CAM retainers, maxillary and mandibular alginate impressions were taken from a male volunteer (aged 35) to produce plaster models from super-hard dental stone (Hinrizit, Ernst Heinrichs GmbH; Goslar, Germany). The models were then digitized by use of a desktop scanner (Ortho X, Dentaurum; Ispringen, Germany) and the derived STL data were used to order the CAD/CAM retainers from the respective manufacturer (Fig. 1). The Twistflex retainers were bent on conventional plaster models.

**Table 1:** List of relevant information for all retainers investigated: manufacturer, product name, material composition, and manufacturing process. *CAD/CAM* computer-aided design/computer-aided manufacturing

	Manufacturer	Product name	Material composition of retainer alloy (%)	Manufacturing process
1	Ormco (Orange, CA, USA)	“Respond” archwire	Stainless-steel alloy 304 (carbon: 0.08; chromium: 10.8/20.0; nickel: 8/10.5; magnesium: 2.0; silicon: 1.00; rest: iron)	Bending
2	Ortholize (Nienhagen, Germany)	No specific product name	Cobalt-chromium alloy (cobalt: 60; chromium: 28; wolfram: 9; silicon 1.5; magnesium, nitrogen, niobium, iron: all < 1)	CAD/CAM (laser melting)
3	Fachlabor Klee (Frankfurt, Germany)	“3D Swiss Retainer”	Grade-5 titanium (aluminum: 5.5; vanadium: 3.5; iron, oxygen, nitrogen, carbon, hydrogen: all < 1; rest: titanium)	CAD/CAM (milling)
4	CA Digital (Hilden, Germany)	“Memotain”	Nitinol (nickel: 55; titanium: 45; oxygen, nitrogen, carbon: all < 1)	CAD/CAM (laser cutting)

Because some of the CAD/CAM retainers are post-processed and a uniform diameter can therefore not be determined, the volume of the retainers could not be calculated geometrically. Hence, the volume was determined by dividing the mass by the density for each included retainer. Density values were provided by the manufacturers. The weight of the retainers was measured to four decimal places using an analytical balance (R180D, Sartorius Research; Goettingen, Germany).

### In vitro MRI scans and quantification of artifact volumes and diameters

**Fig. 2:**
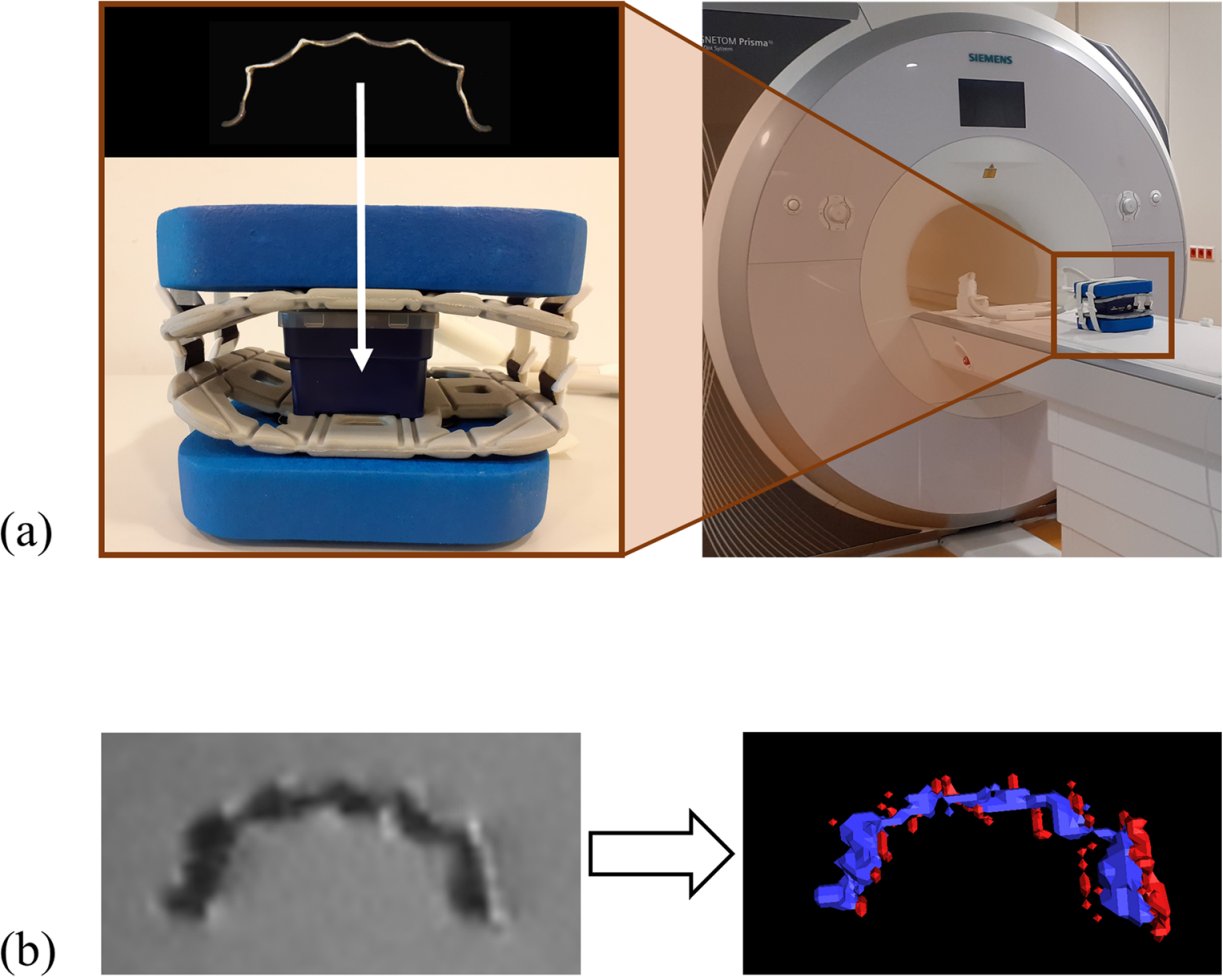
**a**, **b** Workflow for MRI measurement of retainers and subsequent quantification of artifact volumes. **a** Retainers were embedded in agar in plastic boxes. These boxes were then placed into a 16-channel multipurpose coil for in vitro MRI measurement at 3 T. **b** The primary image dataset is shown on the left, in which signal loss artifacts as well as the retainer itself appear hypointense (dark), and pile-up artifacts appear hyperintense (bright). After image acquisition, 3D volumes of hypointense (blue) and hyperintense regions (red) were defined by segmentation, as shown in the volume rendering on the right. Finally, the artifact volume was obtained by adding the volumes of hypointense and hyperintense areas and subtracting the retainer volume. MRI, magnetic resonance imaging; CAD/CAM, computer-aided design/computer-aided manufacturing

Each retainer was embedded in agar gel (Select Agar™, ThermoFisher Scientific; Waltham, MA, USA) in a cuboid plastic box. Next, in vitro MRI measurements were performed using a 3-Tesla (3 T) MRI system (MAGNETOM Trio TIM, Siemens Healthineers; Erlangen, Germany) with a 16-channel multipurpose coil (Variety, Noras MRI products; Hoechberg, Germany). For image acquisition, a T1-weighted, isotropic SPACE (sampling perfection with application-optimized contrasts using different flip-angle evolution) sequence optimized for 3D imaging of the craniomaxillofacial area was used [16]. Sequence parameters were as follows. Matrix: 256 × 256; field of view: 175 mm × 175 mm; voxel size: 0.68 mm × 0.68 mm × 0.68 mm; number of sections: 192; repetition time: 800 ms; echo time: 26 ms; bandwidth: 501 Hz/pixel; slice orientation: coronal; phase-encoding direction: right-to-left; number of averages: 2; echo train length: 63; GRAPPA (generalized autocalibrating partial parallel acquisition) acceleration factor: 2; time of acquisition: 6:59 min. Artifact volumes (AV) were quantified by means of semi-automated segmentation using Amira software (Version 6.4.0, ThermoFisher Scientific) as described elsewhere [36]. This standardized procedure enabled separate 3D identification of signal loss and core and pile-up artifacts. The artifact volume was obtained by adding the volumes of signal loss and pile-up artifacts and subtracting the retainer volume. The ratio of artifact volume to retainer volume (AV/RV ratio) was also calculated. Artifact diameters were measured perpendicular to the retainers’ longitudinal axes at the point with the largest diameter size. The workflow for in vitro MRI measurements and subsequent quantification of artifact volumes is shown in Fig. 2.

## Results

**Fig. 3:**
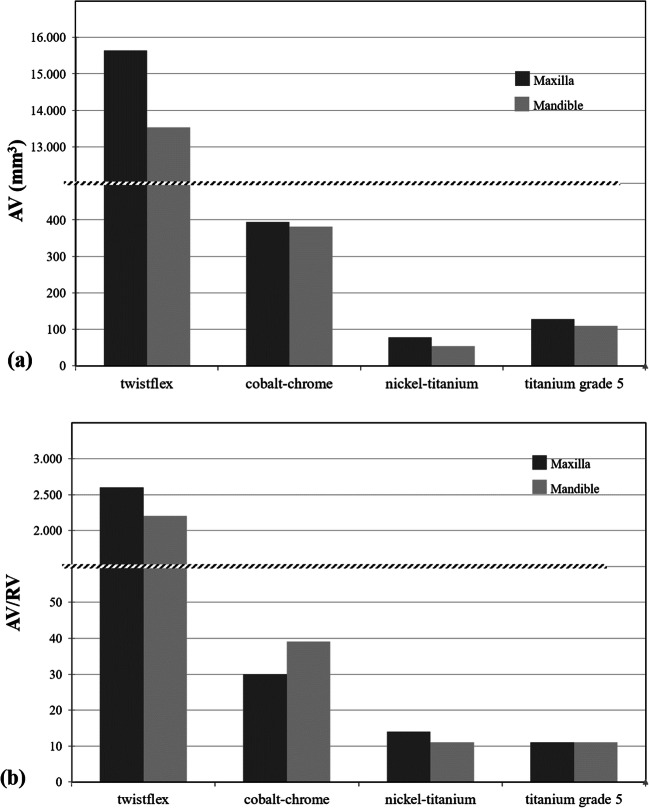
**a**, **b** Total artifact volume (AV; **a**) and corresponding ratio of artifact volume to retainer volume (AV/RV; **b**) for all retainers investigated. Twistflex retainers caused the largest artifact volumes and highest AV/RV ratios. Among the CAD/CAM retainers, the largest artifact volumes and highest AV/RV ratios were observed for cobalt–chromium CAD/CAM retainers. Grade-5 titanium and nickel–titanium CAD/CAM retainers resulted in the smallest artifact volumes and lowest AV/RV ratios. CAD/CAM, computer-aided design/computer-aided manufacturing

**Fig. 4:**
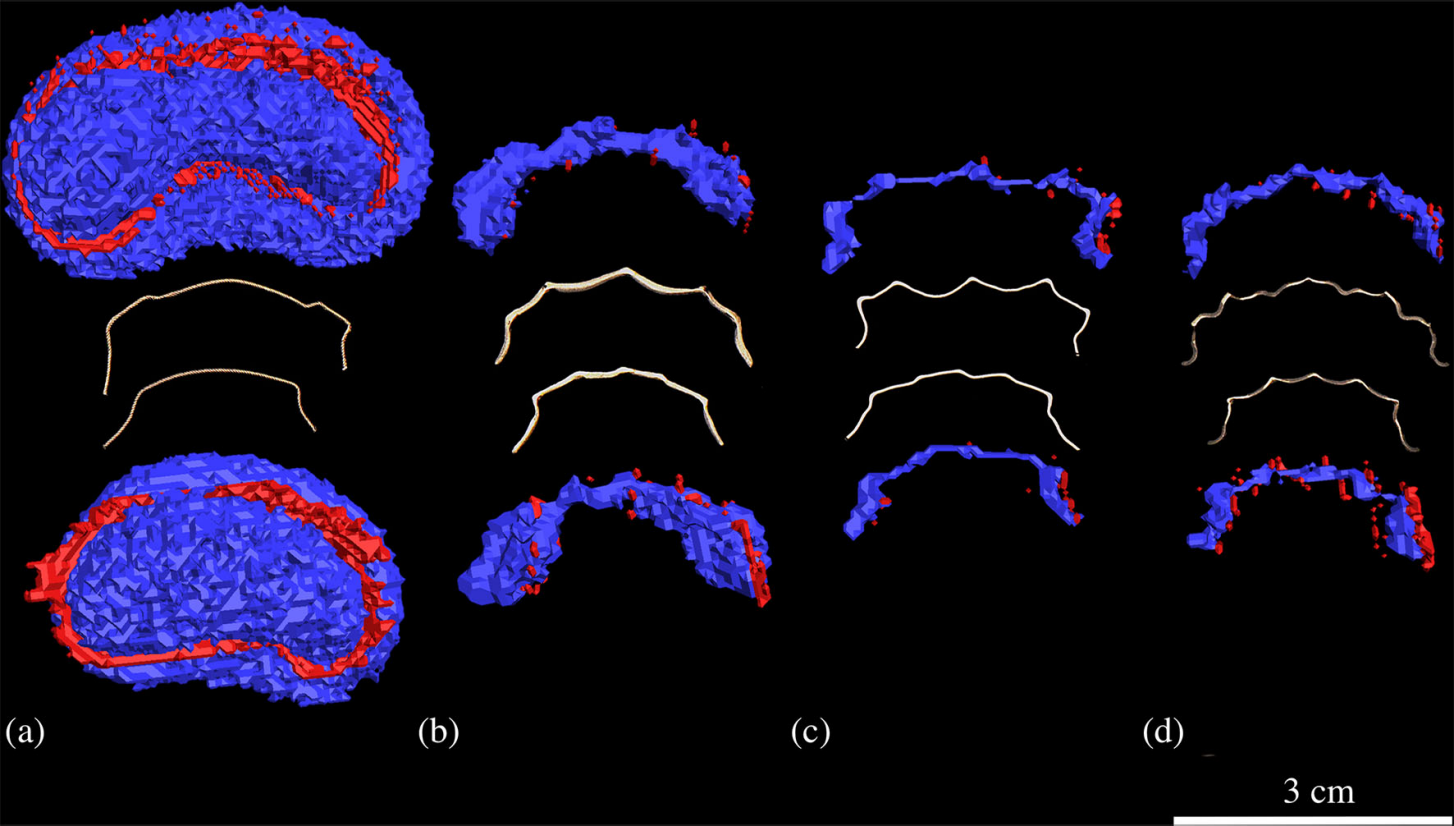
**a*–*d** Retainers with segmented representation of retainer and corresponding artifacts. Hypointense areas (signal loss artifacts and retainer itself) are in blue and hyperintense areas (pile-up artifacts) are in red. A reference line 3 cm in length is inserted on the bottom right. **a** Twistflex (stainless-steel alloy)*.* **b** Cobalt–chromium. **c** Nickel–titanium. **d** Grade-5 titanium

All four retainers caused susceptibility artifacts in the maxilla and mandible to a varying extent (Figs. 3 and 4). The largest artifact volumes were caused by the Twistflex retainers; total artifact volumes were 15,642 mm^3^ in the maxilla (AV_max_) and 13,530 mm^3^ in the mandible (AV_mand_), of which 246 mm^3^ (1.6%) and 315 mm^3^ (2.3%), respectively, were pile-up artifacts. The AV/RV ratio for the maxilla (AV/RV_max_) was 2235, compared with an AV/RV ratio for the mandible (AV/RV_mand_) of 2602. Maximum artifact diameters, measured perpendicular to the longitudinal axis of the retainers, were 32 mm in the maxilla and 28 mm in the mandible.

Among the CAD/CAM retainers tested, the largest artifact volumes were recorded for the cobalt–chromium CAD/CAM retainers (although these volumes were still much smaller than those observed for Twistflex). Total artifact volumes were 394 mm^3^ (AV_max_) and 381 mm^3^ (AV_mand_), of which pile up-artifacts were 12 mm^3^ (3.0%) and 38 mm^3^, respectively. The corresponding ratios of artifact volume to retainer volume were 31 (AV/RV_max_) and 39 (AV/RV_mand_). Artifact diameters perpendicular to the longitudinal axis of the retainers reached maximum values of 9 mm (maxilla) and 8 mm (mandible).

For the grade-5 titanium CAD/CAM retainers, AV_max_ was 126 mm^3^, and AV_mand_ was 110 mm^3^. The corresponding pile-up-artifacts were 17 mm^3^ (maxilla) and 43 mm^3^ (mandible). AV/RV_max_ and AV/RV_mand_ ratios were 11. Artifact diameters perpendicular to the longitudinal axis of the retainers were 4 mm for both the maxilla and for the mandible.

The nickel–titanium CAD/CAM retainers caused the smallest total artifact volumes of all three CAD/CAM retainers, with an AV_max_ value of 78 mm^3^, and an AV_mand_ value of 54 mm^3^, of which 9mm^3^ (11.5%) and 8mm^3^ (14.8%) were pile-up artifacts, respectively. The AV/RV_max_ ratio was 14, compared with the AV/RV_mand_ ratio of 12. Measurements perpendicular to the long axes of the retainers revealed maximum artifact diameters of 3 mm in both the maxilla and mandible*.*

## Discussion

Five-stranded, stainless-steel Twistflex retainers are widely used in orthodontic treatment; recommended by Zachrisson et al. on the basis of over 20 years’ experience, they can be regarded as the clinical reference standard [37]. As the digitalization of orthodontics has progressed, new CAD/CAM retainers made from different materials and using different manufacturing procedures have been introduced [24, 25]; however, the effect of CAD/CAM retainers on MRI imaging has not been investigated so far. In this study, 3D volumes of MRI artifacts caused by three novel CAD/CAM retainers made from cobalt–chromium, grade-5 titanium, and nickel–titanium were determined for the first time and compared with artifacts for the clinically established stainless-steel Twistflex retainer. Importantly, all investigated CAD/CAM retainers caused substantially smaller artifacts compared with Twistflex, with the lowest artifact volumes observed for nickel–titanium and grade-5 titanium. These results indicate that novel CAD/CAM retainers are promising in terms of minor impairment of image quality in head/neck and dental MRI.

A methodological strength of our study is that the quantification of MRI artifacts caused by novel CAD/CAM retainers was based on a reliable, semiautomatic, threshold-based segmentation protocol [36]. This not only enabled 2D recording of artifact diameters like in other in vitro studies of dental materials [27, 29, 38, 39] but also enabled standardized 3D quantification of artifact volumes. To determine artifact volumes, we used an isotropic SPACE sequence that has proved useful for high-resolution, 3D MRI of the craniomaxillofacial area in vivo [16]. Importantly, the image-acquisition parameters remained unchanged from those used in vivo, in order to apply an MRI technique in accordance with realistic clinical conditions.

In our study, Twistflex retainers caused the largest artifacts by far. This is because they are made from stainless steel. Several studies of other appliances such as brackets or arches have already shown that stainless steel causes substantially larger artifacts than nickel–titanium or titanium [20, 33, 40]. One study analyzed MRI artifacts caused by a similar stainless-steel, triple-stranded Twistflex retainer [34]. It is important to note, however, that a different artifact quantification method was used in that study. Specifically, Shalish et al. evaluated MRI artifacts only qualitatively, based on a simple four-scale score of distortions and artifacts in different anatomical regions using a human skull. In contrast, we evaluated the artifacts quantitatively by determining the artifact volumes and diameters in vitro based on a standardized procedure [36], thus providing the basis for a quantitative comparison with artifacts caused by CAD/CAM retainers. Our results showed that Twistflex retainers caused artifacts with diameters of up to 32 mm. This suggests that these artifacts may exceed the dentoalveolar region, which is supported by previous studies [34].

All CAD/CAM retainers caused substantially smaller artifacts (AV up to 200 times smaller) than the Twistflex retainers. Among CAD/CAM retainers, the largest artifacts were observed for cobalt–chromium CAD/CAM retainers. No other study has examined cobalt–chromium retainers before; this is mainly because cobalt–chromium for retainers have only become of interest in the course of CAD/CAM manufacturing due to its material properties [41]. Several studies have examined other cobalt–chromium appliances such as brackets, tubes, or implant superstructures with regard to their artifact behavior in MRI [27, 33, 38, 42, 43], but only a few of these used the same field strength (3 T) as our study for artifact measurement [33, 43]. In our study, the diameters of artifacts caused by cobalt–chromium CAD/CAM retainers were 9 mm (maxilla) and 8 mm (mandible) which is approximately three times smaller than those caused by the cobalt–chromium brackets studied by Blankenstein et al. (coronal: 28.7 mm; axial: 25.2 mm). Although this comparison is restricted by the use of different sequences (our study: SPACE; Blankenstein et al.: gradient echo sequence) as well as different amount and shape of the material, it seems apparent that, whereas artifacts caused by cobalt–chromium CAD/CAM retainers may not exceed the dentoalveolar region, artifacts caused by cobalt–chromium brackets do, at least for conventional gradient echo sequences. This is of clinical relevance, particularly in the context of newly emerging dental-specific MRI methods that enable diagnosis of dentoalveolar disorders [2–6]. For example, dental MRI has gained growing attention in endodontics, including for in vivo assessment of pulp pathologies [44, 45] and differential diagnosis of periapical inflammatory processes [10, 46]. Although the quite small artifacts caused by cobalt–chromium retainers might be only partially relevant for general medical diagnostics, these artifacts could nonetheless impair diagnosis of dental or periodontal pathologies.

The grade-5 titanium CAD/CAM retainers produced artifact volumes that were less than one-third of those caused by the cobalt–chromium CAD/CAM retainers. A recent study of implant superstructures by Hilgenfeld et al. already showed that artifact volumes for cobalt–chromium can be up to ten times larger than those for titanium [43]. The quantitative effect of metals on artifact formation can therefore vary between different appliances of different sizes. Hence, it is difficult to draw conclusions and make predictions about artifacts based on studies of other appliances, even if the materials are the same. In another in vitro study, Blankenstein et al. examined artifacts caused by a twisted titanium retainer (titanium retainer wire, three-strand twisted, Dentaurum), which can be considered the conventional counterpart to the CAD/CAM version investigated in the present study [33]. Whereas we found artifacts with a diameter of 4 mm for the CAD/CAM version, Blankenstein et al. recorded no visible artifacts for the conventional twisted version. These discrepancies are most likely due to the MRI techniques used. Blankenstein et al. used a spin echo sequence and a gradient echo sequence and applied each sequence at 1.5 and 3 T. Importantly, slice thickness in their study was 6 mm for both sequences, which is larger than the maximum artifact diameter of 4 mm observed by us. In comparison, the sequence applied in our study had an isotropic resolution of 0.7 mm. These important differences may explain the discrepancies between our results (very small but visible artifacts) and those of Blankenstein et al. (no visible artifacts). In this regard, it must also be borne in mind that modern 3D MRI sequences, particularly in the field of dento-maxillo-facial imaging, are typically characterized by an isotropic voxel edge length of ≤ 1 mm [9, 15, 47–50]. Accordingly, it is beneficial to use such sequences for in vitro quantification of artifact volumes.

Nickel–titanium CAD/CAM retainers produced the lowest artifacts; however, analysis of the AV/RV ratio of the different CAD/CAM retainers shows that this result is mainly due to the physical volume of the material. If both materials had the same physical volume, the artifacts produced by grade-5 titanium CAD/CAM retainers would actually be slightly smaller than those produced by nickel–titanium CAD/CAM retainers, which is in line with the results of a previous study [33]. From a clinical perspective, however, this point is of limited relevance because the differences in artifact size are too small to warrant a reduction in the diameter of grade-5 titanium CAD/CAM retainers, whose design is primarily intended to minimize complication rates during the period of lifetime wear. Overall, both grade-5 titanium and nickel–titanium CAD/CAM retainers caused very small MRI artifacts and will therefore most likely not result in a relevant impairment of diagnostic image quality in the head and neck region, and even in the dentoalveolar area. Therefore, from the perspective of MR imaging, grade-5 titanium and nickel–titanium CAD/CAM retainers might constitute a substantial improvement to the Twistflex as the current clinical reference standard.

When interpreting the results of this study, several limitations should be considered. First, this study focused on exact quantification of MRI artifacts caused by retainers. Accordingly, it was necessary to perform the study in vitro. However, this means we are unable to draw specific conclusions regarding the in vivo implications of our results. The potential limiting effect of artifacts on diagnosis must therefore be verified by further in vivo studies. In addition, we used a 3-T MRI sequence for artifact measurement that was previously developed and successfully used in an in vivo setting for the application of MRI-based cephalometry and determination of artifacts caused by dental implants [16, 43]. However, MRI artifacts are affected by several variables, particularly by different field strengths and sequences [51]. Thus, applying different MRI techniques will result in different absolute artifact volumes and diameters from those recorded in this study. Further studies are required to examine retainer-induced artifacts caused by different MRI sequences and field strengths.

## Conclusions

Based on this in vitro MRI study, which used a high-resolution, 3D SPACE sequence at 3 T, the following conclusions can be drawn:

Conventional stainless-steel Twistflex retainers cause large artifacts that may exceed the dentoalveolar region. In comparison, all three assessed CAD/CAM retainers caused substantially smaller artifacts that are likely to have a less pronounced effect on image quality in vivo.

The artifacts caused by cobalt–chromium CAD/CAM retainers might limit diagnosis of dentoalveolar disorders by MRI. By contrast, nickel–titanium and grade-5 titanium CAD/CAM retainers caused very small artifacts. This is not only advantageous for head/neck imaging but also for detailed visualization of adjacent dental and periodontal structures.

Overall, the results of this study indicate that novel CAD/CAM retainers are promising in terms of only slightly impairing the image quality of head/neck and dental MRI, with the smallest artifact volumes observed for nickel–titanium and grade-5 titanium. To draw specific conclusions regarding the in vivo implications of our results, however, clinical studies must now follow. Because most retainers remain in place for the patient’s entire lifetime, and MRI scans involving inserted retainers are thus very common, these findings are of clinical relevance for both orthodontists and radiologists.
